# Use of short message service in at-home COVID-19 patient management

**DOI:** 10.1186/s12916-020-01863-9

**Published:** 2020-12-16

**Authors:** Paul Loubet, Christian Czeschan, Matthieu Sintes, Albert Sotto, Didier Laureillard

**Affiliations:** 1 Department of Infectious and Tropical Diseases, CHU Nîmes, Univ Montpellier, 1 Place du Pr Robert Debré, 30029 Nîmes Cedex 9, France; 2 Inserm U1047, University of Montpellier, Nîmes, France; 3 Information Technology Department, CHU Nîmes, Univ Montpellier, Nîmes, France; 4 Pathogenesis and control of Chronic Infections, Inserm, Etablissement Français du Sang, University of Montpellier, Montpellier, France

**Keywords:** Short message service, COVID-19, Follow-up, Outpatients

## Abstract

**Background:**

Mobile health innovations are well adapted for ambulatory coronavirus disease 2019 (COVID-19) patients who risk clinical deterioration at home during the second week of illness.

**Methods:**

A short message service (SMS) communication program was implemented by French physicians to monitor COVID-19 patients after discharge from outpatient or emergency care. The aim of the SMS tracking is to advise patients about their need for medical reassessment if reporting worsening of COVID-19 symptoms.

A follow-up via SMS to all confirmed positive patients in the Nîmes area (France) was established. Every morning, patients received four follow-up questions. Daily responses were converted to green, orange or red trees, analysed in real time by physicians. “Red” patients were called immediately to check their condition and organise transfer to hospital if needed. “Orange” patients were called within two hours to verify whether the specific instructions following the SMS had been followed.

**Results:**

From March 21 to June 30, 2020, 1007 patients agreed to sign up to the SMS tracking, 62% were women and the mean age was 41.5 years (standard deviation (SD) 16.0). During follow-up, 649 (64%) became “orange” and 69 (7%) “red”. Ten patients were directly admitted to the Infectious Diseases Department during their follow-up due to clinical worsening, all but one as a result of SMS alerts and subsequent telephone assessment by physicians.

**Conclusion:**

SMS tracking platforms could be useful as an early warning system to refer patients with worsening clinical status to hospital-based care or additional clinician advice.

## Background

The first non-imported coronavirus disease 2019 (COVID-19) cases were reported in France on February 24, 2020 [1], and the first detected case in the Gard department, south-eastern France, was hospitalised in the Infectious Diseases Department (IDD) of Nîmes University Hospital (CHU Caremeau) on March 2, 2020.

At that time, the diagnostic strategy was to screen all patients with symptoms suggestive of COVID-19 and close contacts of confirmed COVID-19 patients. Screening was performed in the “Ambulatory COVID-19 Screening Centre” and the Emergency Department of the hospital, whereby suspected patients underwent clinical examination and reverse transcriptase polymerase chain reaction (RT-PCR assay) for detecting severe acute respiratory syndrome coronavirus 2 (SARS-CoV-2) on nasopharyngeal samples. Some reports describe clinical deterioration during the second week of illness [2]; thus, follow-up of non-hospitalised, confirmed cases was considered essential. The national lockdown and the risk of transmission made face-to-face consultations logistically difficult.

## Methods

We therefore offered follow-up through a short message service (SMS) to all confirmed positive outpatients and discharged patients from 21 March. Physicians from the IDD developed the SMS tracking platform in collaboration with the Information Technology Department of the Nîmes University Hospital, France. All confirmed patients were called by an IDD physician who relayed the result of the RT-PCR test, explained hygiene and isolation instructions and offered inclusion in the SMS tracking platform. Consenting patients were included in the platform and follow-up started the following day for 14 days.

Every morning at 08:45, patients received the following questions:

“You’re being followed for COVID-19. As agreed, please answer these 4 questions, if possible before 10:30 am. Are you ready? If so, send [1]”.”

“Question 1: Has your condition worsened since yesterday? (No (Send [1]), Moderately (Send [2]), A lot (Send [3])).

Question 2: Do you have trouble breathing? (No (Send [1]), Moderately (Send [2]), Very much, while doing very little (Send [3]))

Question 3: Can you easily manage your daily life (washing, dressing, and eating)? (Yes (Send [1]), No, not at all (Send [2]))

Question 4: Do you feel able to continue home confinement? (Yes (Send [1]), No, not at all (Send [2]))

Questions were chosen to be easily understandable and to cover the different aspects of COVID-19 symptoms and the related isolation (clinical, psychological and practical). The questionnaire took less than 5 min per day to complete.

An automated reminder was sent if no response was received by 12:00. The tracking platform generated a web page with each row representing a patient integrated in a separate dashboard distinct from patients’ medical chart. Daily responses were converted to green, orange or red trees, analysed daily in real time from 08:00 to 20:00. One infectious diseases physician dedicated 50% of their working hours to the inclusion of patients, colour checking and contacting the orange/red patients.

Patients with only “1” responses were displayed in green and received the following message: “Thank you for participating. Your condition is stable or improving. Follow-up continues at home. Continue to adhere to strict containment guidelines. See you tomorrow.”

If there was at least one “2” response but no “3”, patients were displayed in orange and the following message was sent: “Thank you for participating. It seems to us that your condition requires re-evaluation by your general practitioner today to continue follow-up at home. Please continue to adhere to the strict containment guidelines. See you tomorrow.”

If there was at least one “3” response, patients were displayed in red and the following message was sent: “Please dial 15 [emergency medical assistance service] promptly so that your condition can be assessed by an emergency and/or infectious diseases specialist.”

“Red” patients were called immediately to check the patient’s condition and organise transfer to hospital if needed. “Orange” patients were called within 2 h to see if the specific instructions following the SMS had been followed.

We were rapidly able to offer the inclusion of patients into the tracking platform to nine other ambulatory COVID-19 centres in the Gard department: two secondary general hospitals and seven general practitioner groups caring for suspected and confirmed COVID-19 patients.

General practitioners were informed of the existence of the SMS tracking system and how to join via e-mail from the ARS (Regional Health Agency).

## Results

From March 21 to June 30, 2020, five patients (all healthcare workers) refused to be included and 1007 patients agreed to sign up to the SMS tracking, 62% were women and the mean age was 41.5 years (standard deviation (SD) 16.0). Of these, 994 (99%) had at least 1 day of follow-up and 850 (85%) more than 5 days of follow-up. The mean length of follow-up was 13.2 days (SD 5.4).

**Fig. 1:**
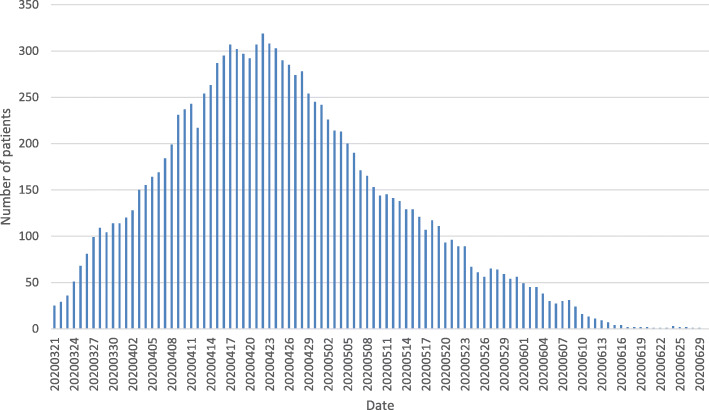
Number of COVID-19 confirmed patients followed in the SMS tracking platform every day from March 21 to June 30, 2020

Figure 1 displays the number of people who used the SMS system at any point during the study period.

During follow-up, 649 (64%) became “orange” and 69 (7%) “red”, of whom 30 progressed from “orange” to “red” and 39 from “green” to “red”. We found no differences in age and sex between the 688 patients who became red or orange (64% of women, mean age 41.7 years, SD 15.2) and those who stayed green (60% of women, mean age 40.8 years, SD 18.2) (Table 1).

**Table 1:** Characteristics of the 1007 COVID-19 confirmed patients followed in the SMS tracking platform every day from March 21 to June 30, 2020, according to their follow-up status

	Total ***N*** = 1007	Stayed “green” ***n*** = 688	Moved to “orange” and/or “red” ***n*** = 319	***p*** value
Percentage of women (95% CI)	62 (59–65)	60 (56–64)	64 (59–69)	0.16^a^
Mean age, years (SD)	41.5 (5.0)	40.8 (18.2)	41.7 (15.2)	0.64^b^
Follow-up, days (SD)	13.2 (5.4)	11.2 (5.4)	14.4 (5.2)	0.19^b^

^a^Fisher’s exact test

^b^Student’s *t* test

Ten patients were directly admitted to the IDD during their follow-up due to clinical worsening. Nine were evaluated as “orange” and one as “red”. Admission resulted from SMS alerts and subsequent telephone assessment by physicians. The mean time from SMS tracking inclusion and hospital admission was 3 days (SD 0.5).

## Discussion

The main limitation of our study is that the impact assessment of our SMS tracking platform was not possible because we were unable to create a control group due to the high participation rate and the early launch of the platform in the epidemic, without data for the cases that occurred prior to the launch.

Mobile health interventions, including SMS, increase access to care and enhance the efficiency of health service delivery in different conditions [3–5]. Patients with moderate COVID-19 disease can often isolate and be monitored at home to avoid overcrowding in healthcare facilities and save hospital beds for more severe cases. SMS, compared to traditional phone call, provides low cost, instant transmission of information and better accessibility compared to phone apps, especially in older patients who are particularly affected by the severe forms of COVID-19.

Most mobile health interventions in the context of COVID-19 have been developed for screening patients (self-reported symptoms tracking app in healthy volunteers may help identify incidence of the disease, as well as novel symptoms [6, 7]) or to help contact tracing.

## Conclusion

We share here one example of a physician-monitored SMS to connect with patients with COVID-19 after discharge from outpatient or emergency care that was quickly developed and highly acceptable to patients and clinicians. SMS tracking platforms could be useful as an early warning system to refer patients with worsening clinical status to hospital-based care or additional clinician advice.

## Data Availability

Data are available upon reasonable request to the corresponding author.

## Abbreviations

COVID-19: Coronavirus disease 2019
IDD: Infectious Diseases Department
RT-PCR: Reverse transcriptase polymerase chain reaction
SARS-CoV-2: Severe acute respiratory syndrome coronavirus 2
SMS: Short message service
